# Iodine in Neuralgia

**Published:** 1857-08

**Authors:** 


*Iodine in Neuralgia*—W. G. Thornton, M. D., of Texas, reports a Case (in the New Orleans Medical News and Hospital Gazette,) of Neuralgia of the head, successfully treated by the application of the Tincture of Iodine. " After the failure of the ordinary remedies," he says, " I applied it, and its effects were almost immediate. I had not finished its application before the pain began to diminish, and soon ceased entirely, and has not since returned."

This application he followed by the internal use of Syrup of Sarsaparilla and Iodide of Potassium.

